# Electrical stimulation for the treatment of spinal cord injuries: A review of the cellular and molecular mechanisms that drive functional improvements

**DOI:** 10.3389/fncel.2023.1095259

**Published:** 2023-02-03

**Authors:** Ryan M. Dorrian, Carolyn F. Berryman, Antonio Lauto, Anna V. Leonard

**Affiliations:** ^1^Spinal Cord Injury Research Group, School of Biomedicine, The University of Adelaide, Adelaide, SA, Australia; ^2^IIMPACT in Health, University of South Australia, Adelaide, SA, Australia; ^3^School of Science, Western Sydney University, Penrith, NSW, Australia

**Keywords:** spinal cord injury, peripheral nerve stimulation (PNS), epidural electrical stimulation (EES), functional electrical simulation (FES), neuroplasticity, neuroinflammation

## Abstract

Spinal cord injury (SCI) is a devastating condition that causes severe loss of motor, sensory and autonomic functions. Additionally, many individuals experience chronic neuropathic pain that is often refractory to interventions. While treatment options to improve outcomes for individuals with SCI remain limited, significant research efforts in the field of electrical stimulation have made promising advancements. Epidural electrical stimulation, peripheral nerve stimulation, and functional electrical stimulation have shown promising improvements for individuals with SCI, ranging from complete weight-bearing locomotion to the recovery of sexual function. Despite this, there is a paucity of mechanistic understanding, limiting our ability to optimize stimulation devices and parameters, or utilize combinatorial treatments to maximize efficacy. This review provides a background into SCI pathophysiology and electrical stimulation methods, before exploring cellular and molecular mechanisms suggested in the literature. We highlight several key mechanisms that contribute to functional improvements from electrical stimulation, identify gaps in current knowledge and highlight potential research avenues for future studies.

## 1. Introduction

Traumatic spinal cord injury (SCI) is a devastating neurological injury that significantly impedes motor, sensory, and autonomic functions. Additionally, an estimated 53% of individuals with SCI experience chronic neuropathic pain that is often refractory to treatment (Burke et al., 2017). Such deficits drastically alter the individual’s lifestyle, and along with personal and social factors, are associated with a diminished quality of life (Post and Noreau, 2005). Given the limited efficacy of current treatments, there is a critical need to develop novel, effective interventions that improve outcomes.

Current clinical management of SCI involves immediate stabilization of the vertebral column and early spinal cord decompression, followed by extensive physical rehabilitation (Fehlings et al., 2017). Beyond this, treatment options that promote significant neurological and functional recovery remain limited (Khan and Ahmed, 2022). Extensive research efforts have developed several novel interventions currently under investigation. One potential treatment is electrical stimulation, which has shown promising functional improvements when delivered epidurally over the spinal cord or to peripheral nerves.

Electrical stimulation has a long history of therapeutic use, despite a paucity of mechanistic understanding. Electrical stimulation was first used in ancient Rome, where shocks from torpedo ray fish were used for treating headaches and gout (Kellaway, 1946; Khan and Ahmed, 2022). However, there was little understanding of the role of electricity in the nervous system until the late 1700s, when Luigi Galvani, aided by his wife Lucia, successfully conducted electricity through a frog’s nerves (Galvani, 1791). In 1803, Galvani’s nephew, Giovanni Aldini, used electrical stimulation to stimulate the muscles and “reanimate” the corpse of an executed man (Aldini, 1803). Later that century, du Bois-Reymond and Bernstein made the first recording of an action potential (Bernstein, 1868), the latter proposing that the permeability of nerves to potassium ions was responsible for a negative resting membrane potential (Bernstein, 1902). Hodgkin and Huxley later challenged this hypothesis and extensively characterized the ionic basis of the action potential, for which they won a share of the 1963 Nobel Prize (Hodgkin and Huxley, 1952).

The greater understanding of electricity’s role in nervous system functions eventually led to its adoption in clinical medicine. Liberson (1961) delivered stimulation via surface electrodes placed over the peroneal nerves to control foot drop in hemiplegic patients. Concomitant developments in pain mechanisms saw the emergence of the gate control theory (Melzack and Wall, 1965). Wall and Sweet later demonstrated components of this theory by inducing pain relief via electrical stimulation of the infraorbital nerves (Wall and Sweet, 1967). Further research has identified the response of glial cells to electrical stimulation and expanded the gate control theory to a complex model of pain processing that includes all levels of the nervous system and all homeostatic systems (Shiao and Lee-Kubli, 2018; Finnerup et al., 2021). Also Shealy et al. (1967) demonstrated that electrical stimulation delivered through the epidural space could alleviate diffuse chest and abdominal pain. Together, these studies showed that both epidural and peripheral electrical stimulation could offer relief from neuropathic pain and contributed to the development of the neuromodulation research field (Gildenberg, 2006).

Neuromodulation strategies have since shifted focus toward achieving functional recovery following SCI. Epidural electrical stimulation (EES), peripheral nerve stimulation (PNS) and functional electrical stimulation (FES), among other forms of stimulation, have been applied post-SCI with promising results. Functional improvements from these devices range from weight-bearing locomotion to regaining sexual function and neuropathic pain relief (Harkema et al., 2011). While the potential benefits of electrical stimulation post-SCI are apparent, the cellular and molecular mechanisms that drive these functional improvements remain unclear (Eisdorfer et al., 2020; Choi et al., 2021; Furlan et al., 2021). Understanding these mechanisms would enable the optimization of stimulation devices and parameters, maximizing their clinical efficacy (James et al., 2018). Furthermore, there is an expanding field of research combining electrical stimulation with other therapeutic interventions (Fadeev et al., 2021). Thus, understanding the mechanisms of electrical stimulation may allow for more targeted combinatorial therapies with synergistic effects that further improve outcomes (Zheng et al., 2020).

This review aims to outline the potential cellular and molecular mechanisms of electrical stimulation post-SCI that may drive improvements to motor function, autonomic functions and neuropathic pain. We highlight key research areas for future studies to explore and identify gaps in knowledge that may need addressing.

## 2. SCI pathophysiology

Understanding SCI pathophysiology is necessary to evaluate the potential mechanisms by which electrical stimulation may improve outcomes. SCI pathophysiology is biphasic, consisting of the primary injury and the subsequent secondary injury cascade (Rowland et al., 2008).

### 2.1. Primary injury mechanisms

The primary injury is the initial mechanical spinal cord damage due to the traumatic event. Contusion plus persistent compression is the most common form of primary injury seen clinically (Tator, 1995), but laceration, transection, distraction, or contusion plus transient spinal cord compression may also occur (Oyinbo, 2011). Regardless of the form, primary injury causes immediate tissue damage that impedes ascending and descending spinal cord pathways (Alizadeh et al., 2019; Anjum et al., 2020). Unfortunately, this occurs instantaneously and is considered irreversible. However, the subsequent secondary injury cascade is a clear target for therapeutic intervention (Silva et al., 2014).

### 2.2. Secondary injury mechanisms

**FIGURE 1:**
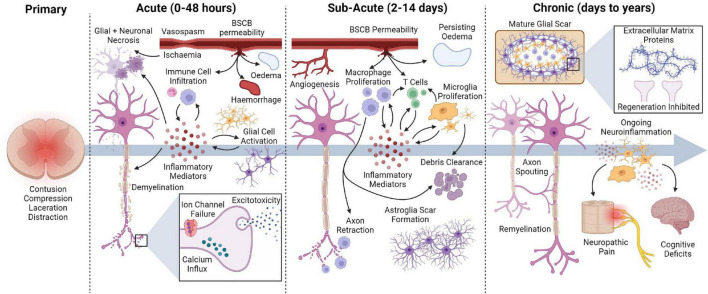
Overview of secondary injury events following traumatic SCI, highlighting key pathophysiological events that occur throughout the acute, subacute, and chronic stages of injury.

The secondary injury is a complex cascade of pathophysiological events that expands tissue damage beyond the initial trauma and worsens outcomes. Secondary injury begins immediately following the primary injury and persists through acute (0–48 h), subacute (48 h to 14 days), and chronic stages (days to years) (Figure 1) (Anjum et al., 2020). Understanding these events is crucial, as the delayed nature of the secondary injury cascade makes these events potentially amenable to electrical stimulation therapies.

#### 2.2.1. Acute secondary injury

The acute secondary injury phase immediately follows the traumatic event and involves significant necrotic cell death, axonal dysfunction, inflammation, and vascular processes. Shortly after injury, Ca^2+^ and Na^+^ imbalances occur via multiple mechanisms (Li and Stys, 2001; Liu et al., 2011; O’Hare Doig et al., 2017; Illes, 2020). These imbalances can cause cell death via lipid peroxidation, free radical species production, and glutamate excitotoxicity (Li and Stys, 2000; Liu et al., 2011; Ahuja et al., 2017). Glutamate excitotoxicity contributes to oligodendrocytes cell death, which increases axonal vulnerability and impedes signal transduction (Li and Stys, 2001; Totoiu and Keirstead, 2005).

The SCI induces a significant inflammatory response that persists indefinitely. As resident immune cells within the spinal cord, microglia are among the first responders to injury, along with astrocytes and peripheral immune cells (neutrophils and macrophages) (Oyinbo, 2011). Despite having beneficial roles, these cells cause significant tissue damage by releasing various toxic molecules that induce DNA damage, lipid peroxidation and, ultimately, cell death (Hellenbrand et al., 2021; Zivkovic et al., 2021).

Spinal cord vasculature is also compromised post-SCI, leading to severe hemorrhaging, Blood-Spinal Cord Barrier (BSCB) disruption, and vasospasm (Ahuja et al., 2017). These events can lead to edema formation, potentially raising intrathecal pressure and reducing spinal cord perfusion (Saadoun et al., 2008; Leonard et al., 2014).

#### 2.2.2. Subacute secondary injury

Secondary injury persists into the subacute phase of SCI, with vascular and inflammatory events continuing to extend tissue damage. Angiogenesis occurs within the injured spinal cord between 3 and 7 days post-SCI (Figley et al., 2013). However, BSCB permeability and edema remain present (Yao et al., 2021), facilitating the recruitment of blood-derived monocytes to the injury (Beck et al., 2010). In the spinal cord, macrophages can physically interact with axons, causing them to retract from the injury site (Horn et al., 2008). Macrophages and microglia continue to propagate neuroinflammation, which is necessary for clearing tissue debris but can also cause extensive tissue damage (Kwon et al., 2004).

In response to the ongoing tissue damage, a glial scar begins to form and section off the injury site to prevent further lesion expansion. Astrocytes proliferate and become hypertrophied between 3 and 5 days post-SCI (Yang et al., 2020). They then accumulate around the lesion core and extend their processes to create a protective barrier against further injury spread (Orr and Gensel, 2018).

#### 2.2.3. Chronic secondary injury

The chronic phase of secondary injury involves the maturation of the glial scar, which contains the injury site (Yang et al., 2020). Within the scar, macrophages primarily populate the lesion epicenter, while microglia migrate toward the perilesional border (Hellenbrand et al., 2021). While beneficial for preventing further injury spread, extracellular matrix proteins within the glial scar and myelin-associated inhibitors (Nogo, MAG, and OMgp) can prevent neurite outgrowth and impede recovery (Geoffroy and Zheng, 2014; Alizadeh et al., 2019). While remyelination and neuroplasticity can occur, individuals with chronic SCI experience minimal functional recovery (Ahuja et al., 2017).

Neuroinflammation continues indefinitely after SCI and may contribute to several complications, including cognitive decline and neuropathic pain (Hains and Waxman, 2006; Faden et al., 2016). Neuropathic pain post-SCI involves complicated peripheral, central and supraspinal mechanisms that are yet to be fully elucidated (Shiao and Lee-Kubli, 2018). These mechanisms have recently been reviewed in considerable detail (Finnerup et al., 2021) and consist of complex alterations in neuronal circuitry, microglia, astrocytes, and various other pathways that may contribute to central sensitization.

### 2.3. Summary of SCI pathophysiology

The SCI pathophysiology involves several complicated processes set in motion by the primary injury. As these events can extend tissue damage and worsen outcomes, understanding the influence of electrical stimulation on secondary injury processes is vital.

## 3. Electrical stimulation following SCI

Electrical stimulation is a promising intervention for individuals with SCI, with its potential based on several key observations. Firstly, evidence of residual supraspinal connections below the injury site is observed post-SCI, even in individuals classified with “clinically complete” injuries (Sherwood et al., 1992). This suggests that the sub-lesioned neural circuitry still receives supraspinal inputs, but these residual inputs are insufficient to produce function. Despite this, the neural circuitry below the lesion is generally preserved and maintains functional properties when externally stimulated (Edgerton et al., 2004). Thus, it is possible to deliver electrical stimulation below the injury site to influence activity. Finally, the lumbosacral central pattern generator (CPG) can respond to external electrical stimulation to facilitate locomotion (Dimitrijevic et al., 1998). Thus, electrical stimulation may restore movement in individuals with SCI, even if the sub-lesioned circuitry cannot respond to supraspinal inputs.

**FIGURE 2:**
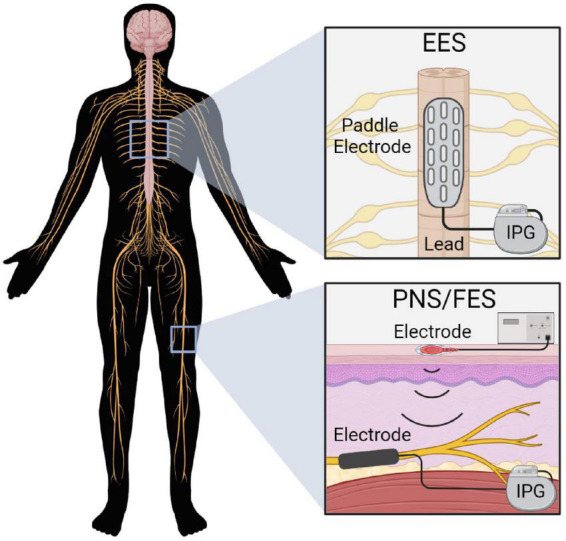
Electrical stimulation devices commonly used post-SCI. EES delivers stimulation over the spinal cord via a paddle electrode array that receives power from an implanted pulse generator (IPG). PNS and FES deliver stimulation to the target nerve via implanted electrodes connected to an IPG, or via percutaneous or transcutaneous electrodes.

There are several techniques and devices used to stimulate the nervous system post-SCI. Of these strategies aiming to influence the sub-lesioned spinal cord, EES, PNS, and FES have shown promising results (Figure 2).

### 3.1. Epidural electrical stimulation (EES)

Epidural electrical stimulation involves the application of electrical stimulation over the spinal cord via an implanted paddle electrode array. Stimulation is generally applied over the lumbosacral spinal cord to promote locomotion; however, cervical EES may also be used depending on the desired outcome (Malone et al., 2021). To deliver EES, a paddle electrode array is surgically implanted over the spinal cord via a laminectomy, with electrode positioning confirmed via x-ray, fluoroscopy and electrophysiology (Calvert et al., 2019a). Current electrode arrays typically consist of 16 individual electrodes that can be activated in specific patterns to produce the desired outcome.

Shealy et al. (1967) first used EES in 1967 to relieve chronic pain. Following this, the technique received significant attention for studying the locomotor CPG (Calvert et al., 2019a). Iwahara et al. (1992) demonstrated that stimulation (C4–C8 and L1–L6) could induce locomotor stepping patterns in decerebrate cats. Later, Dimitrijevic et al. (1998) utilized lumbosacral EES in SCI patients to elicit rhythmic locomotor activity. This study provided evidence for a lumbosacral CPG within humans. Harkema et al. (2011) would later demonstrate the therapeutic potential of EES following SCI. In an individual with chronic, motor-complete and sensory incomplete SCI (AIS B), Harkema et al. (2011) demonstrated that EES and intense rehabilitation facilitated full weight-bearing locomotion. This significant study led to the broader adoption of EES post-SCI.

The EES has since shown promising results in numerous clinical trials. The expansion of Harkema’s 2011 study replicated these results, restoring voluntary motor control in four additional participants – two of whom had AIS A (motor and sensory complete) injuries (Angeli et al., 2014). Subsequent studies by Harkema’s group have progressed the extent of functional recovery achievable with EES. In 2015, they utilized lumbosacral EES to achieve full weight-bearing standing in four participants with AIS A or B injuries (Rejc et al., 2015). Later, Angeli et al. (2018) facilitated independent over-ground locomotion in two participants with AIS B injuries through EES and an intensive rehabilitation program. After observing gains in bladder, sexual and thermoregulatory functions (Harkema et al., 2011), Harkema’s group have also explored the effects of EES on other physiological systems. Aslan et al. (2018) demonstrated that EES could prevent drops in blood pressure when standing in participants with cardiovascular dysregulation post-SCI. Also in Herrity et al. (2018) optimized EES stimulation parameters to improve voiding efficiency in an individual with AIS B SCI. These stimulation parameters were then applied to four additional participants who also improved in voiding efficiency but to a lesser extent (Herrity et al., 2018). Nonetheless, Herrity’s subsequent study demonstrated significant improvements in bladder capacity, compliance and detrusor pressure with activity-based recovery training and EES that was not optimized for bladder function (Herrity et al., 2021). Thus, there appear to be off-target effects from motor-focused EES, which may be enhanced by optimizing stimulation parameters.

Several other studies corroborate the findings of Harkema’s group. Gill et al. (2018) utilized lumbosacral EES and multimodal rehabilitation to facilitate independent standing and aided overground stepping in a man with complete functional loss post-SCI. Later, Darrow et al. (2019) verified that EES could restore voluntary movements in two female participants with complete AIS A injuries. Additionally, both participants reported improved bladder and bowel function, and one achieved orgasm, supporting the suggestion of off-target effects with EES (Darrow et al., 2019). Similarly to Harkema, Courtine’s group has demonstrated vast improvements in motor function with EES. Spatiotemporal EES restored overground walking within 1 week of stimulation, and locomotion function improved with EES-mediated rehabilitation. After rehabilitation, participants demonstrated meaningful increases in walking function, and two participants could transition from sitting to standing, and crutch-assisted walking without active EES (Wagner et al., 2018). Building upon this, Courtine’s group developed an electrode array optimized for the recovery of motor functions, which enabled standing, walking, cycling, swimming and trunk control in three chronically paralyzed individuals. After intense activity-specific neurorehabilitation, these activities were transferrable to community settings (Rowald et al., 2022). Cervical EES is less studied but also appears promising, improving hand control and strength in two patients with cervical injuries (Lu et al., 2016).

### 3.2. Limitations of EES

Despite the promising improvements to motor and autonomic functions, EES has some limitations. Notably, electrode implantation requires an invasive surgical procedure that, although generally free of complications, may compromise spinal stability and risk infections (Calvert et al., 2019b). An alternative that addresses these issues is transcutaneous spinal cord stimulation, which is provided externally without needing a surgical procedure. While transcutaneous stimulation has shown promising improvements in voluntary movement, muscle strength and muscle function, these studies remain preliminary (Megía García et al., 2020). Further research must demonstrate efficacy in controlled studies that account for potential placebo effects before transcutaneous stimulation is a viable alternative (Megía García et al., 2020). Nonetheless, developing less-invasive alternatives to EES is a clear direction for improvement.

The EES devices may also require further optimization to maximize efficacy. Stimulation parameters are often patient-specific and optimizing activation patterns is time-intensive and may delay rehabilitation (Calvert et al., 2019b). Further, stimulation has generally been delivered in a continuous pattern. However, continuous EES pulses can disrupt natural proprioceptive signals vital for recovering locomotor function (Formento et al., 2018). Spatiotemporal EES can preserve these natural inputs and has shown promising results in human trials (Wagner et al., 2018). Hence, future EES studies may benefit from implementing this paradigm. Finally, SCI studies generally use the same electrodes as chronic pain studies. As such, they are not optimized for recovering motor function, which is often the primary outcome (Calvert et al., 2019b). Rowald et al. (2022) recently addressed this by developing an optimized electrode array for functional recovery post-SCI. Further use of this electrode array may improve efficacy in future studies.

Limitations in EES study designs may also require consideration. Clinical EES trials are currently restricted to a few groups worldwide that often use small sample sizes with similar participant characteristics. Hence, it is difficult to determine how applicable the results are to broader demographics. Preliminary results examining the generalizability of EES are auspicious, with Darrow et al. (2019) demonstrating efficacy in a small cohort of women with chronic SCI. Nonetheless, future research must demonstrate the benefits of EES in larger sample sizes and broader patient demographics. A further caveat specific to clinical trials is effective blinding, given that both researchers and participants are aware of functioning stimulation. Whilst current outcomes are primarily focused on demonstrating efficacy, whereby blinding isn’t imperative, meeting regulatory requirements for clinical trials moving forward may be problematic (Choi et al., 2021).

Finally, participants in EES studies generally receive intense physical rehabilitation that likely exceeds that typically provided post-SCI (Calvert et al., 2019b). For example, in Rowland and colleagues’ study, participants received 1–3 h of personalized rehabilitation, 4–5 days each week, over 5 months (Rowald et al., 2022). This accumulates to 80–300 h of rehabilitation. In contrast, the SCIRehab study found that individuals received an average of 55.3 h of rehabilitation during inpatient programs (Taylor-Schroeder et al., 2011). This discrepancy in rehabilitation time must be considered when interpreting results from EES studies. For individuals outside of clinical trials, there are barriers to physical rehabilitation, including a lack of access and transport to rehabilitation centers (Gorgey, 2014). It may not be feasible for individuals who receive EES implants in typical settings to undertake the physical therapy regimes currently utilized in clinical trials, potentially impacting outcomes. Hence, optimizing EES treatments and rehabilitation regimes may be a crucial future step to improve accessibility.

### 3.3. Peripheral nerve stimulation (PNS)

Electrical stimulation can also be applied directly to the peripheral nerves below the injury. There are two main applications of peripheral nerve stimulation: for therapeutic purposes (PNS) and functional purposes (FES; reviewed in section 3.5).

Similarly to EES, PNS historically stems from neuropathic pain management after Wall and Sweet induced neuropathic pain relief via infraorbital nerve stimulation (Wall and Sweet, 1967). The greater commercial availability of PNS devices facilitated its wider application across several pain conditions, including amputee pain, back pain and headache/facial pain (Chakravarthy et al., 2016). PNS has also seen significant applications post-SCI for managing neuropathic pain (Celik et al., 2013), along with attempts to restore motor (Lee et al., 2015) and autonomic functions (Kamboonlert et al., 2021).

**FIGURE 3:**
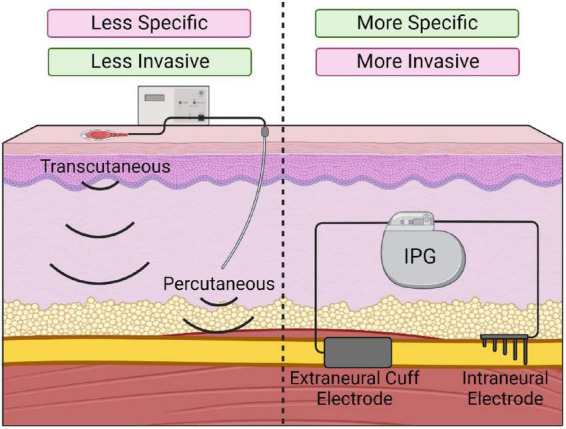
Various types of peripheral nerve stimulation devices. Transcutaneous and percutaneous stimulation **(Left)** offer temporary and minimally invasive stimulation but are less specific. Implanted electrodes (**Right**; extraneural or intraneural) offer more specific nerve stimulation but require invasive implantation.

Multiple PNS devices have been developed and vary in invasiveness and specificity of nerve stimulation (Figure 3). Transcutaneous and percutaneous devices deliver stimulation through surface and needle electrodes, respectively, offering accessible and temporary PNS (Trevathan et al., 2019). Alternatively, long-term stimulators can be surgically implanted by dissecting the target nerve and securing an electrode array. The device is then anchored to the tissue using sutures and is connected to an internal or external pulse generator (Harsh et al., 2019). Implanted stimulators can encompass the outside of the nerve via spiral, cuff or flat paddle interface designs. Alternatively, more specific and invasive devices penetrate the nerve to facilitate the targeted activation of nerve fascicles (Günter et al., 2019).

The PNS following SCI has shown several improvements to motor, autonomic, and neuropathic pain outcomes. In a 2015 study with 22 patients within 6 months of injury, percutaneous stimulation (100 Hz, 30 min daily, 5 days per week for 6 weeks) was applied unilaterally to the common peroneal nerve while participants attempted to contract their target muscles. This short-term PNS protocol ameliorated several motor axon dysfunctions caused by SCI (Lee et al., 2015). Bilateral tibial nerve stimulation (10 hz, 20 min) significantly improved urodynamic parameters in 15 participants with neurogenic detrusor overactivity post-SCI (Kamboonlert et al., 2021). Furthermore, a single session of lower limb transcutaneous nerve stimulation (100 Hz, 30 min) significantly reduced spasticity in SCI participants (Sivaramakrishnan et al., 2018). Low-frequency transcutaneous stimulation (4 Hz, 30 min for 10 days) has also demonstrated significant reductions in neuropathic pain in 33 patients with SCI (Celik et al., 2013). Preclinical rodent investigations have shown similar efficacy. In a rat model of severe T11-12 contusion, sacral nerve stimulation via a needle electrode (10 hz, 2–3 mA, 15 min each day for 14 days) significantly improved motor and autonomic functions, evidenced by increased BBB open-field score at 16 days post-SCI, shortened the time to fecal efflux and improved feces quality and texture (Zhu et al., 2020). Additionally, 1 h of sciatic nerve stimulation (20 hz) following a rodent T8 dorsal column lesion can promote axonal outgrowth across the lesion site (Udina et al., 2008; Goganau et al., 2018). These studies demonstrate the promising efficacy of PNS following SCI to improve outcomes beyond motor function, representing meaningful improvements in quality of life for people with SCI.

### 3.4. Limitations of PNS

Similarly to EES, there are several limitations with PNS. Like EES, appropriate stimulation parameters for PNS are often patient-specific and differ significantly depending on the intended outcome. Unlike EES, several PNS devices are available and deliver stimulation via differing methods. While this provides greater flexibility, the weigh-off between specificity and invasiveness must be considered (Trevathan et al., 2019). Implanted electrodes are often recommended for long-term use as they are generally well-tolerated post-SCI (Delianides et al., 2020; Freeberg et al., 2020), and result in more precise nerve activation with lower stimulation parameters (Ho et al., 2014). However, this involves a surgical procedure with inherent risks, and device implantation can potentially cause nerve damage due to suture placement or through scarring and adhesions (Slavin, 2011). Additionally, electrode migration or breakage may warrant device replacement (Slavin, 2011).

These limitations are being addressed by developing smaller and less invasive PNS devices. Sliow et al. (2019) developed the “graft-antenna,” a gold strip incorporated on a chitosan scaffold that facilitates minimally invasive implantation through laser-tissue bonding and wireless PNS via magnetic stimulation. The “graft-antenna” has shown efficacy following nerve transection but has not been investigated post-SCI. Also Trevathan et al. (2019) developed the “injectrode” – a prepolymer element with conductive properties that is injected around a target nerve to facilitate stimulation. While these novel devices require further characterization in SCI models, they may provide less invasive stimulation methods for PNS that could aid clinical usability.

The lack of descending drive for PNS interventions may limit treatment efficacy by reducing the supraspinal influence on function. Several techniques are under investigation to address this limitation. Paired associative stimulation (PAS) combines PNS with transcranial magnetic stimulation of the motor cortex (Suppa et al., 2017). While outside this review’s scope, PAS has shown promising results, including voluntary motor function recovery and diminished pain (Shulga et al., 2016). Alternatively, patients receiving PNS can be prompted to visualize or attempt to move the stimulated muscle to replicate descending drive, as implemented by Lee et al. (2015). Descending drive can also be achieved by pairing PNS with a functional task – known as functional electrical stimulation therapy.

### 3.5. Functional electrical stimulation (FES)

Functional electrical stimulation (FES) utilizes PNS to induce the muscular contractions necessary for the individual to complete a functional task. Similarly to PNS, transcutaneous, percutaneous or implanted electrodes deliver electrical stimulation for FES (Marquez-Chin and Popovic, 2020). Stimulation is targeted to the peripheral nerves rather than the muscles themselves, as the lower power requirements for nerve stimulation reduce the risk of tissue damage and patient discomfort (Peckham and Knutson, 2005).

The FES was first used in 1961 to control foot drop after stroke (Liberson, 1961) but became increasingly utilized for SCI thereafter. FES can be used as a neuro-prosthetic system that allows functional control of the paralyzed musculature within community settings. Indeed, FES systems have been developed to facilitate standing, stepping, bladder and bowel function, erection and ejaculation, and reaching and grasping activities, among others (Ragnarsson, 2008). Alternatively, FES can be used therapeutically to promote long-lasting functional improvements (Marquez-Chin and Popovic, 2020). This clinical application of FES, known as FES therapy, utilizes FES to aid more standard rehabilitative functions such as walking, cycling and rowing exercises, or task-based therapies such as reaching and grasping (Luo et al., 2020).

Regardless of the activity performed, FES has shown promising improvements in patient function post-SCI. A recent phase II trial of 27 chronic incomplete motor SCI patients examined the efficacy of a 16-week body weight-supported treadmill training program combined with lower limb FES training. Participants in the FES-assisted walking training group significantly improved spinal cord independence measures (SCIM) mobility sub-scores compared to the aerobic and resistance training control group (Kapadia et al., 2014). In 24 individuals within 6 months of SCI, FES combined with conventional occupational therapy significantly improved grasping function across a battery of voluntary grasping compared to conventional occupational therapy alone (Popovic et al., 2011). Recently, Atkins and Bickel (2021) showed that FES therapy could significantly increase muscle size, function, and metabolism. Cardiovascular benefits have also been reported, although a recent study using FES rowing did not observe a significant decrease in cardiovascular disease (Solinsky et al., 2021). While more research may be required to elucidate cardiovascular outcomes, improvements to motor function highlight the potential of FES post-SCI.

### 3.6. Limitations of FES

Many limitations outlined for PNS also apply to FES. Many FES studies use transcutaneous stimulation via temporary surface electrodes that can be difficult and time-consuming to place correctly. Implanted systems can address this issue, but these require invasive implantation and risk device malfunction. As with PNS, developing simpler and less invasive stimulation devices will likely aid the uptake of implanted FES devices. A challenge with FES systems is that the stimulation induces rapid muscle fatigue, likely due to the reversed recruitment of muscle fibers with electrical stimulation (Dolbow et al., 2014). Electrical stimulation primarily activates large-diameter fibers, while natural stimulation recruits small fibers first. The reversal of recruitment order may result in faster muscle fatigue, impacting the length and efficacy of FES rehabilitation. While this remains a challenge, several strategies to reduce fatigue have been trialed with variable success (Ibitoye et al., 2016).

## 4. Cellular and molecular mechanisms of electrical stimulation

Electrical stimulation post-SCI via EES, PNS or FES has shown promising results; however, our understanding of the cellular and molecular mechanisms driving these improvements is lacking. Several studies (Table 1) have suggested multiple potential mechanisms, including:

**TABLE 1:** Details of key studies examining the mechanisms of electrical stimulation.

References	Study details	Key outcome	Stimulation parameters	Acute/immediate functional outcomes	Functional outcomes at conclusion	Adverse events	Analysis methods for mechanisms	Mechanism explored
**Epidural electrical stimulation**								
Angeli et al. (2014)	Clinical study: –4 Males 23.8–32.8 years old. –2.3–4.2 years post-SCI. –Injury levels: C7, C6–C7, T5, and T5–T6. –AIS grade: –A (2) and B (2).	Motor function	16 electrode array at L1 – S2 –25 Hz or 30 Hz. –0.8–9 V. –Participant specific. –450 μs pulse width. –Continuous stimulation. –1 h daily.	3/4 participants could voluntarily execute movements within 4–11 days of stimulation. Final participant showed voluntary movement after 7 months of stimulation but was not assessed earlier.	Voluntary motor function continued to improve in all participants with long-term rehabilitative training and EES.	None reported	Electrophysiology	Descending neuroplasticity: –Modulation of motor output in response to visual and auditory cues.
Angeli et al. (2018)	Clinical study: –4 participants 22–32 years old. –2.5–3.3 years post-SCI. –Injury levels: C5, T1, and T4 (2). –AIS grade: –A (2) and B (2)	Motor function	16 electrode array at L1 – S2 –20–50 Hz. –1–5.7 V. –Participant specific. –450 μs pulse width. –1–2 training sessions daily with 1 h of stimulation.	Not reported	Long term training and stimulation facilitated intentional overground walking in 2/4 participants. All four participants achieved independent standing and trunk stability. –Execution of walking only with active EES and participant’s intention to walk.	Drainage of surgery site (1). Ankle oedema (1). Hip fracture during training (1).	Motor and sensory exam.	Descending neuroplasticity: –Walking only occurred with intent of participant.
Kathe et al. (2022)	Clinical study: –9 males 23–53 years old. –1 year, 3 months – 14 years, and 3 months post-SCI. –Injury levels: T3, T4, T6, and unclear for 6 participants. –AIS grade: A (2), B (1), C (5), and D (1). –Preclinical study: –Male or female mice severe contusion (95 kdyne) T8/T9 SCI. –Endpoint at 30 min or after 4 weeks rehabilitation	Motor function. –Mechanisms (preclinical).	Clinical study: 16 electrode array: specify 5-6-5 medtronic paddle lead or a custom design (ONWARD medical) –parameters personalized. –Preclinical study: Electrodes at L2 and S1 Continuous stimulation: –40 Hz, 0.2 ms pulses, and 50–300μA. High frequency burst stimulation: –10 ms busts of 0.2 ms pulses. –50–300 μA. –600 Hz. –30 Hz modulating frequency.	All participants immediately improved/regained walking with robotic interface support. Participants exerted volitional control over stepping amplitude.	5 months of EES rehab improved weight-bearing capacities, outdoor walking with EES and assistive device.	None reported	Neuroimaging (PET). Electrophysiology. Tract tracing. Immunohistology. Single nucleus RNA sequencing. Spatial Transcriptomics.	Descending neuroplasticity: –Decreased neuronal activity within lumbar spinal cord. –Identified neuronal population necessary for walking with EES.
Kinfe et al. (2017)	Clinical study: –12 people with Failed Back Surgery Syndrome (6 females and 5 males; 44–76 years old). –Blood samples obtained at baseline and at 3 months of stimulation.	Pain	16 contact paddle lead at Th8-9 –40 Hz burst rate. –500 Hz intra-burst rate. –1 ms pulse width. –0.15–1.6 mA intensity (participant specific). –Three months of stimulation.	Not reported	3 months of burst stimulation significantly decreased back and leg pain intensity (visual analog scale).	No serious adverse events reported. –Temporary skin irritation at implanted pulse generator site (3).	Enzyme-linked immunosorbent assays (ELISA)	Neuroinflammation: –Elevated serum IL-10 expression.
Asboth et al. (2018)	Preclinical study: –Female rat severe contusion (255.5 kdyne) T8/9 SCI. –Male or female C57BL/6 mice severe contusion (90 kdyne) T8-9 SCI. –Endpoint at 12 weeks post-SCI.	Motor function	Electrodes at L2 and S1 –Continuous stimulation at 40 Hz. –0.2 ms pulse width. –100–300 μA. –Training 6 days per week for 40 min. –Combinatorial treatment: EES paired with agonists to serotonergic and dopaminergic receptors (electrochemical neuromodulation).	Electrochemical neuromodulation immediately restored involuntary locomotion on treadmill but not voluntary.	100% of rats with electrochemical neuromodulation and 88% of rats with EES only regained weight-bearing locomotion. Trained rats adapt limb kinematics for different tasks.	None reported	Behavioral testing. Electrophysiology. Tract tracing. Optogenetics. Immunohistology.	Descending neuroplasticity: –Cortico-reticulo-spinal networks facilitate descending control.
Ghorbani et al. (2020)	Preclinical study: –Male rat moderate contusion (150 kdyne) T10 SCI. –Endpoint at 15 days post-SCI.	Mechanisms	Electrodes at upper injury site (T10) –Subthreshold stimulation at 100 Hz, 0.1 ms. 0.3–0.6 mA. –1 h stimulation daily for 14 days.	Functional outcomes not assessed.	Functional outcomes not assessed.	None reported.	Immunoblotting. Immunohistology	Neurotrophic factors: –Elevated BDNF in homogenized spinal cord samples.
Li et al. (2020)	Preclinical study: –Female rat contusion (25 mm 20 g weight drop) T10 SCI. –Endpoints at 7 and 28 days post-SCI.	Motor function	Custom Array at T10 – T13 –Stimulation at 90% motor threshold. –50 Hz. –200 μs pulse width, 0.045 mA. –30 min stimulation per day for 1 week.	Significantly improved BBB score at 7 days post-SCI.	Significantly improved BBB scores at 14, 21, and 28 days post-SCI.	None reported.	Semi quantitative RT-PCR. Immunoblotting. Immunofluorescence. Histology.	Glial cells: –Reduced oligodendrocyte and myelin loss.
Sivanesan et al. (2019)	Preclinical study: –Paclitaxel-induced peripheral neuropathy study in male rats. –Endpoint at day 30.	Pain	Quadripolar medtronic SCS electrode. –T13 to L1 spinal cord level. –50 Hz, 0.2 ms, constant current, and 80% motor threshold. –6–8 h daily for 14 days.	Stimulation before and during paclitaxel administration alleviated the development of neuropathic pain.	Neuropathic pain relief extended for at least 2 weeks after stimulation.	Spinal cord injury from implant. Poor lead placement Damaged electrodes	RNA-seq	Neuroinflammation: –Upregulating of genes involved in inflammatory processes, particularly astrocytes and microglia.
Stephens et al. (2018)	Preclinical study: –Chronic constriction injury of sciatic nerve in male and female rats. –Endpoint at 39 days post injury.	Pain	Quadripolar medtronic SCS electrode. –T13 to L1 spinal cord level. –50 Hz, 0.2 ms, constant current, and 80% motor threshold. –120 min/session, twice per day for 3 days.	Peak neuropathic pain relief within 60–90 min of commencing stimulation. Withdrawal responses returned to baseline within 30 min of ceasing stimulation.	Long term outcomes not assessed.	Impaired motor function after implantation (3).	RNA-seq.	Neuroinflammation: –Upregulating of genes involved in inflammatory processes.
Thornton et al. (2018)	Preclinical study: –Female rat T7/T8 complete transection injury. –Endpoint at 6–7 months post-SCI.	Motor. –Mechanisms.	Electrodes at L2 and S1 –40 Hz at 95% of threshold. –Training for 20 mins per day, 3 days per week for 6 months. –Combinatorial treatment: cellular transplant of OECs or FBs.	Short term outcomes not assessed.	No difference in function between OEC and FB implanted animals either with or without EES.	None reported	Immunohistology.	Axonal regeneration: –Greater presence of NF and 5-HT positive axons into lesion.
Vallejo et al. (2020)	Preclinical study: –Male rat spared nerve injury model. –Endpoint after 48 h of stimulation.	Pain	Four electrode SCS lead (Heraeus medical). –Low rate stimulation: –50 Hz, 150 μs pulse width, 0.03–0.09mA (70% motor threshold). High rate stimulation: –1,200 Hz, 50 μs pulse width, 0.02–0.010 mA (70% motor threshold). DTMP stimulation: –Multiplexed charges in 20–1,200 Hz range, 500 μs max pulse width, 0.03–0.10 mA (70% motor threshold).	Significantly improved mechanical hypersensitivity with all stimulation forms. DTMP significantly improved hot and cold thermal hypersensitivity.	Long term outcomes not assessed.	None reported	RNA-seq	Neuroinflammation –Upregulating of genes involved in inflammatory processes. –Decreased GFAP, C1qa, Casp1, and Tal1 expression.
**Peripheral nerve stimulation**								
Perez et al. (2003)	Clinical study: –Twenty health volunteers (13 men and 7 women). Mean age 29.4 years old.	Motor function.	30 min of common peroneal nerve Patterned stimulation: –10 pulses at 100 hz every 1.5 s. –1 ms pulse width. Uniform stimulation: –10,000 pulses total. –150 ms even spacing of pulses.	Patterned stimulation enhanced reciprocal inhibition for at least 5 min after treatment. Effect was temporary and returned to baseline by 20 min.	Long term outcomes not assessed.	None reported.	Electrophysiology.	Local neuroplasticity: –Improved strength of reciprocal inhibition.
Ayanwuyi et al. (2022)	Preclinical study: –Male rat T12 unilateral dorsal column focal demyelination. –Endpoint at 7 or 14 days post-demyelination.	Mechanisms	Sciatic nerve stimulation –20 Hz continuous stimulation for 1 h. –100 ms pulse width, 3 V.	Functional outcomes not reported.	Functional outcomes not reported.	None reported.	Immuno-fluorescence.	Glial cells: –Increased myelin basic protein expression, increased oligodendrocyte numbers. –Neuroinflammation: –Microglia/macrophage polarization to pro-repair phenotype.
Gajewska-Woźniak et al. (2016)	Preclinical study: Healthy male rats	Mechanisms	Tibial nerve –Continuous burst of 3 pulses every 25 ms. –200 μs pulse width. –4 ms inter-pulse interval. –4 min × 20 min daily for 7 days.	Functional outcomes not reported.	Functional outcomes not reported.	None reported.	Retrograde labeling. Electrophysiology Immuno-fluorescence	Local neuroplasticity: –Increased glutaminergic and cholinergic inputs to alpha motoneurons.
Goganau et al. (2018)	Preclinical study: –Female rat C4 dorsal column wire knife lesion. –Endpoint at 4 weeks post-SCI.	Mechanisms. Pain.	Sciatic nerve –20 Hz stimulation. –0.2 ms pulse duration. –1 h stimulation post-SCI.	Electrode placement was not associated with neuropathic pain.	Fine touch did not recover with stimulation.	No adverse effects of pain.	Immunohistology. Tract tracing.	Axonal regeneration: –Increased neurite length after C4 dorsal column injury.
Hahm et al. (2014)	Preclinical study: –Male rat severe contusion (50 mm 10 g weight drop) T12 SCI. –Endpoint at 5 weeks post-SCI.	Spasticity	Bilateral hindlimb stimulation: transcutaneous stimulation at 4 Hz or 100 Hz. –250 μs pulse width. –50% or 90% motor threshold. –30 min single session 5 weeks post-SCI.	High frequency stimulation alleviated spasticity from 20 to 50 min after application.	Long term effects not assessed.	None reported.	Immunohistology.	Neuroinflammation: –Reduced activated microglia expression.
Hayashi et al. (2019)	Preclinical study: –Male rat severe contusion (25 mm 20 g weight drop) T9 SCI. –Endpoint at 1 or 4 weeks post-SCI.	Motor function.	Bilateral hindlimb stimulation –2 Hz at 10 mA. –10 min of stimulation 5 days per week, 4 weeks total.	No improvement to BBB score at 1 week post-SCI.	BBB score significantly higher from 2 to 4 weeks post-SCI for stimulation animals. –Significantly higher inclined plane angle at 4 weeks post-SCI for stimulated animals.	None reported	Histology. TUNEL staining. Immunoblotting. Immunohistology. ELISA.	Neurotrophic factors: –Increased levels of BDNF in spinal cord.
Matsuo et al. (2014)	Preclinical study: –Spared nerve injury in male jcl:ICR mice. –Endpoint 8 days post-injury.	Pain.	Electrodes over L1 to L6 dorsal rami. –Transcutaneous stimulation at 100 Hz. –Sub-motor threshold Intensity for 30 min.	Early stimulation significantly reduced mechanical hyperalgesia from days 3–7 of stimulation. –Significantly reduced thermal hyperalgesia at days 6 and 7.	Late stimulation (1 or 2 weeks post-injury) did not alleviate neuropathic pain.	None reported.	Immunohistology. Immunoblotting Flow cytometry	Neuroinflammation: –Decreased microglia and astrocyte activation and pro-inflammatory cytokines.
Udina et al. (2008)	Preclinical study: –Female rat T8 dorsal funiculus lesion. –Endpoint at 15 weeks post-SCI.	Mechanisms.	Sciatic nerve –20 Hz at 0.02 ms pulse width or 200 Hz stimulation for 25 ms every 250 ms. –72,000 pulses per hour. –2× motor threshold. –0.02 ms pulse width. – 1 h stimulation post-SCI.	Functional outcomes not reported.	Functional outcomes not reported.	None reported.	Tract tracing. ELISA. Immunohistology.	Axonal regeneration: –Increased axonal outgrowth into T8 dorsal column injury. –Neurotrophic factors: –Increase in cAMP in lumbar dorsal root ganglion.
Wenjin et al. (2011)	Preclinical study: –Female rat sciatic nerve transection model. –Endpoint at 1 week post-injury.	Mechanisms.	Sciatic nerve –Continuous 20 Hz stimulation. –100 μs pulse width and 3–5 V. – 1 h of total stimulation.	Functional outcomes not reported.	Functional outcomes not reported.	None reported.	Immuno-fluorescence.	Neurotrophic factors: –Increased levels of BDNF in spinal cord neurons.
Wong et al. (2022)	Preclinical study: –Male rat L5 nerve root ligation. –Endpoint at 7 days after injury.	Pain.	Sciatic nerve stimulation –2, 20, or 60 Hz. –200 μs square wave pulses, pulse trains separated by 8 s. –1–10 mA intensity. –1 h single stimulation session (L5 nerve root ligation model).	2 and 20 Hz stimulation alleviated mechanical and thermal hypersensitivity from day 1 to 7 post-injury.	Long term outcomes not assessed.	None reported.	Immuno-fluorescence. Immunoblotting.	Neuroinflammation: –Decreased microglia and astrocyte density, decreased inflammatory cytokines.
**Functional electrical stimulation**								
Bakkum et al. (2015)	Clinical study: –FES cycling: 9 males 40–64 years old. –10–34 years since SCI. –Injury levels: C3, C6, C7 (2), T1, T6, T8, T9, and T10. –AIS grade: –A (6), B (1), and C (2).	Mechanisms. General health outcome.	FES cycling –Surface electrodes over quadriceps, gluteal and hamstring muscles. –50 Hz stimulation for all muscles. –Adjustable intensity with maximum of 140 mA. –16 weeks exercise program, 18–32 mins cycling time.	Short term outcomes not assessed.	Both FES cycling and control hand cycling improved inflammatory status, visceral adiposity, and metabolic syndrome symptoms.	None reported, but high dropout rate (46%) possible due to time intensive training program.	ELISA	Neuroinflammation: –Reduced CRP, IL-6, and IL-6/IL-10 ratio.
Griffin et al. (2009)	Clinical study: –18 participants (13 male and 5 female) aged 27–56 years old (mean = 40). –1–53 years post-injury (mean = 11). –Injury levels: C4 (5), C5, C8 (2), T2, T3, T4 (3), T5 (2), T6, and T7(2). –5 complete injuries, 13 incomplete.	Motor and sensory score	FES cycling –Surface electrodes over quadriceps, gluteal and hamstring muscles. –50 Hz, <140 mA. –2–3 times weekly, 10 weeks.	Short term outcomes not assessed.	Lower extremity ASIA scores and motor and sensory components of the ASIA test improved with FES training.	None reported.	ELISA	Neuroinflammation: –Reduced CRP, IL-6, and TNF-α expression.
Piazza et al. (2017)	Clinical study: –10 participants (6 males and 4 females) aged 32–72 (mean = 48.1). –Time since injury: 12 to 43 months (mean = 32). –Injury levels: C4 (9) and C6. –AIS grades: C (6) and D (4).	Motor function.	10 min FES cycling –200 Hz stimulation. –1 ms pulse width. –Intensity just below generation of visible muscle contractions.	Increase in H-reflex excitability after stimulation.	Long term outcomes not assessed.	None reported.	Electrophysiology.	Local neuroplasticity: –Improved H reflex.
Becker et al. (2010)	Preclinical study: –Female rat suction-ablation T9 SCI. –Endpoint at 36 or 43 days post-SCI.	Mechanisms	Common peroneal nerve –20 Hz stimulation. –200 μs pulse width and 3V. –Alternated 1 s on/off for each leg. –Stimulation 3 times a day for 1 h	Functional outcomes not assessed.	Functional outcomes not assessed.	None reported.	Immunohistology.	Glial cells: –Increased progenitor cell birth and differentiation into oligodendrocytes.

1. Neuroplastic remodeling
2. The upregulation of neurotrophic factors
3. Influence on glia and neuroinflammation

### 4.1. Principles of electrical stimulation

The basic principles of electrical stimulation of the nervous system stem from Hodgkin and Huxley’s characterization of the action potential (Hodgkin and Huxley, 1952). Within the axon, the resting membrane potential is held around −70 mV. As the axon becomes slightly depolarized and reaches the threshold potential (approximately −55 mV), Na^+^ enters the axon, causing depolarization and action potential propagation. Similarly, electrical stimulation can depolarize the axon to this threshold and induce a bi-directional action potential (Peckham and Knutson, 2005). This idea allows nerves to be stimulated for therapeutic purposes.

Evidence suggests that electrical stimulation primarily activates large-diameter afferent fibers involved in proprioception. Large-diameter fibers are predominantly activated due to their size, allowing for easier stimulation at lower intensities (Peckham and Knutson, 2005). FES and PNS stimulate these afferents within the peripheral nerve; while EES activates large-diameter afferents in the posterior roots (Rattay et al., 2000; Gerasimenko et al., 2006; Capogrosso et al., 2013; Duffell and Donaldson, 2020). As EES, PNS and FES activate similar afferents, there is likely a degree of overlap between the cellular and molecular mechanisms (Duffell and Donaldson, 2020).

**FIGURE 4:**
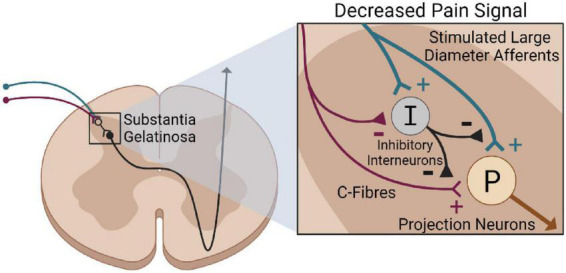
Gate control theory of pain following electrical stimulation. Stimulating large-diameter afferents (blue) via electrical stimulation excites inhibitory interneurons (black) in the substantial gelatinosa. This suppresses the stimulation of projection neurons (brown) via C-Fibers, reducing pain perception.

Activating large-diameter afferents is a potential mechanism driving neuropathic pain relief via electrical stimulation. This idea stems from Melzack and Wall (1965)’s gate control theory of pain. The gate control theory suggests that interneurons in the substantia gelatinosa act as a “gate” that modulates incoming sensory information before central transmitting cells are activated. This gate is influenced by descending input from the brain, and through large and small-diameter afferent fibers (Melzack and Wall, 1965). Activating small-diameter afferents “opens” the gate and excites projection neurons, transmitting a pain signal to the brain. Alternatively, activating large-diameter afferents “closes” the gate by activating interneurons that inhibit ascending pain signals. Therefore, activating large-diameter afferents by electrical stimulation may excite dorsal horn inhibitory interneurons, which suppress painful stimuli (Figure 4) (Strand et al., 2021).

While the gate control theory provides an elegant explanation for pain relief via electrical stimulation, our understanding of neuropathic pain has significantly progressed (Mendell, 2014). Neuropathic pain is a complex condition, and this research field has shifted toward a more multifaceted understanding of mechanisms. For example, the tetrapartite synapse model suggests that a functional unit composed of four factors – the pre and post-synaptic neurons, microglia, and astrocytes – plays a critical role in neuropathic pain (De Leo et al., 2006). Similarly, Finnerup et al. (2021) suggest the involvement of epigenetics, ion channel alterations, immune cell activation, and glial-derived mediators. While the gate control theory may play a role, it is also likely that these alternative mechanisms contribute to neuropathic pain relief via electrical stimulation.

### 4.2. Neuroplasticity

Neuroplastic remodeling within the spinal cord is one of the most explored mechanisms for electrical stimulation. Large-diameter afferents branch extensively in the spinal cord, meaning electrical stimulation can exert modulatory effects at multiple locations. These afferents synapse on alpha-motoneurons in the ventral gray matter, allowing direct influence over motor activity (Guertin, 2013). They can also indirectly engage antagonistic muscles through polysynaptic interneuron connections (Eisdorfer et al., 2020). Thus, large-diameter afferents can influence motor function by activating agonistic muscles and inhibiting antagonists. Proprioceptive afferents can also influence motor activity through their inputs to the CPG. Indeed, stimulating large-diameter afferents can trigger this intrinsic circuit and modulate CPG activity throughout locomotion (Guertin, 2013). Finally, proprioceptive afferents relay sensory information to supraspinal centers through the dorsal column medial lemniscus pathway.

**FIGURE 5:**
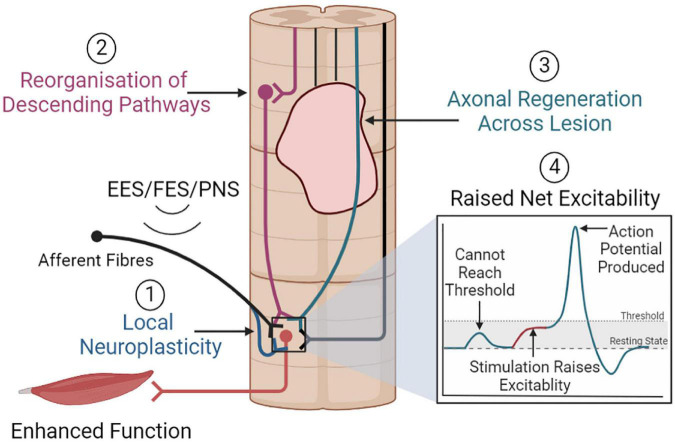
Overview of neuroplastic mechanisms that may promote functional improvements. (1) Electrical stimulation may promote local neuroplasticity, strengthening motoneuron activation from afferent or descending inputs. (2) Stimulation may also promote the reorganization of descending pathways, or (3) promote axonal regeneration, which together could facilitate greater supraspinal control. (4) Stimulation may raise net spinal cord excitability, allowing the sub-lesioned circuitry to respond to weak residual supraspinal inputs and immediately restore function.

As such, numerous sites within the nervous system may be influenced by the stimulation of large-diameter fibers. This may induce neuroplastic spinal cord remodeling via several mechanisms that may ultimately improve function (Figure 5).

#### 4.2.1. Local neuroplasticity

Electrical stimulation may induce local neuroplasticity between afferent fibers, interneurons, and motoneurons (Eisdorfer et al., 2020). Synaptogenesis between these neurons may result in greater motoneuron activation from afferent inputs, improving muscle recruitment. Supporting this, Piazza et al. (2017) found that FES-cycling exercise significantly increased H-reflex excitability in participants with incomplete SCI. While this study was limited by sample size, it suggests that electrical stimulation may modulate this synaptic circuit (Piazza et al., 2017). Simultaneously, electrical stimulation may strengthen the synapse between afferent fibers and inhibitory interneurons within the spinal cord (Eisdorfer et al., 2020). In healthy rats, tibial nerve stimulation (continuous busts of three pulses every 25 ms, 4-min × 20-min daily sessions, 7 days) can significantly increase the number of direct glutamatergic and indirect cholinergic inputs to alpha-motoneurons (Gajewska-Woźniak et al., 2016). Further, patterned stimulation of the common peroneal nerve in healthy human volunteers (100 Hz every 1.5 s) resulted in a significant – albeit temporary – increase in reciprocal inhibition (Perez et al., 2003). Thus, electrical stimulation may improve muscular functions by strengthening afferent inputs onto interneurons and motoneurons, allowing for greater muscle recruitment and more robust inhibition of antagonist muscles.

While reorganization within this local circuit would be beneficial, it is unlikely that this mechanism alone accounts for the improved outcomes observed with electrical stimulation. Indeed, several lines of evidence suggest that supraspinal centers can influence function during electrical stimulation. Angeli et al. (2014) noted that participants with EES could modulate the timing and intensity of motor movements in response to auditory and visual cues, indicating some form of voluntary supraspinal control. Later, Angeli et al. (2018) showed that overground walking with EES only occurred when the participant actively intended to walk. Thus, there appears to be some degree of supraspinal control over motor function in the presence of electrical stimulation. The key role of supraspinal centers in functional recovery with electrical stimulation was demonstrated in Asboth et al. (2018). In a mouse SCI model, combining optogenetic stimulation of the motor cortex with electrochemical neuromodulation (EES with serotonergic and dopaminergic receptor agonists) immediately restored weight-bearing locomotion. However, ceasing motor cortex stimulation prevented locomotion function (Asboth et al., 2018). Thus, descending supraspinal pathways appear vital for functional improvements.

#### 4.2.2. Reorganization of descending pathways

A potential source of descending inputs to the sublesional neural circuitry are residual supraspinal connections that survive the traumatic event and retain connections beyond the lesion. Indeed, it is well established that most clinical SCI cases exhibit a degree of connectivity beyond the lesion site, even in cases classified as motor or sensory complete (Sherwood et al., 1992).

While residual descending pathways exist post-SCI, they are considered “functionally inactive” and unable to produce meaningful movement without interventions (Angeli et al., 2018). It has been suggested that electrical stimulation may raise the net excitability of the spinal cord circuitry to facilitate movement (Harkema et al., 2011). Under normal circumstances in people with SCI, inputs from residual supraspinal connections cannot reach the threshold required to elicit a functional response. However, electrical stimulation may provide a basal level of excitation that allows supraspinal inputs to reach this threshold and influence function. This is likely a key mechanism that facilitates immediate functional improvements with stimulation. However, this does not explain long-lasting functional improvements that persist without stimulation. Indeed, Asboth et al. (2018) noted that 62.5% of rats trained with electrochemical neuromodulation could perform voluntary movements without EES. Similarly, in a chronic, clinically complete SCI individual, long-term activity-based training combined with EES allowed for volitional movement of the lower limbs and independent standing without stimulation (Rejc et al., 2017). As these long-lasting improvements persist after ceasing stimulation, they cannot entirely be attributed to increased net excitability. Rather, electrical stimulation may induce neuroplastic remodeling within the spinal cord, creating a specialized neural circuit that is functionally active and facilitates movement after SCI.

In support of this, Kathe et al. (2022) observed that EES combined with rehabilitation decreased metabolic activity within the lumbar segments in nine participants with various severities of SCI. To investigate further, the group used spatial transcriptomics and single-nucleus RNA sequencing within a mouse model of EES and SCI. This analysis identified a population of excitatory lumbar interneurons, SC^*Vsx*2:*Hoxa*10^, that were particularly responsive to EES post-SCI (Kathe et al., 2022). While neuronal activity decreased within the mouse lumbar spinal cord after stimulation, transcriptional activity within SC^*Vsx*2:*Hoxa*10^ neurons doubled in response to EES and rehabilitation. Further analysis revealed that these neurons exclusively project to the ventral spinal cord, where they establish dense glutamatergic, GABAergic and cholinergic synapses on neurons necessary for locomotion. In addition, analysis revealed that SC^*Vsx*2:*Hoxa*10^ neurons receive input directly from large-diameter afferents that are engaged by electrical stimulation. Hence, these specialized interneurons appear to play a key role in recovering locomotor function with electrical stimulation.

Interestingly, Kathe et al. (2022) noted that SC^*Vsx*2:*Hoxa*10^ neurons receive descending input from reticulospinal neurons. Thus, the supraspinal influence over function during electrical stimulation may be facilitated by the reticulospinal tract (ReST). The ReST originates from the various brainstem nuclei within the reticular formation and makes widespread connections within the spinal cord. The ReST was initially thought to influence axial and proximal limb muscles for posture regulation. However, studies in primate models suggest that the ReST has overlapping connections with the corticospinal tract and may have a significant role in functional recovery post-SCI (Riddle and Baker, 2010).

Asboth et al. (2018) examined the role of the ReST in the recovery from a rodent severe contusion SCI using electrochemical neuromodulation. Electrochemical neuromodulation immediately restored voluntary movement. However, tract-tracing revealed that the contusion completely abolished corticospinal tract projections below the injury site. Alternatively, they found that a subset of neurons within the vestibular nuclei and ventral gigantocellular reticular nuclei (vGi) received motor cortex projections and retained lumbar connectivity. When glutamatergic neurons within the vGi were silenced via a Cre-dependent AAV2/1 vector carrying a G_*i/o*_-specific DREADD (Designer Receptor Exclusively Activated by Designer Drug), functional movements with electrochemical neuromodulation were abolished, highlighting the crucial role of these neurons. Also, rats that received rehabilitation with electrochemical neuromodulation had a threefold increase in the density of vGi neurons below the injury (Asboth et al., 2018). Thus, the brain may retain communication with the sub-lesioned spinal cord via glutamatergic vGi neurons that project through the ReST (Courtine and Sofroniew, 2019). This finding, coupled with the observation that SC^*Vsx*2:*Hoxa*10^ neurons receive ReST input, is significant, and suggests the involvement of both the ReST and SC^*Vsx*2:*Hoxa*10^ neurons in long-term functional recovery with electrical stimulation.

Asboth et al. (2018) also noted that cortico-reticulo-spinal neurons established close connections with propriospinal neurons within thoracic spinal cord segments. Hence, propriospinal neurons may also present a potential pathway that facilitates supraspinal control (Harkema et al., 2011; Angeli et al., 2014). Propriospinal neurons are interneurons contained entirely within the spinal cord. These neurons facilitate communication between spinal cord segments and can act as relays for ascending and descending signals (Flynn et al., 2011). Following SCI, propriospinal circuits can undergo remodeling to facilitate supraspinal control below the injury site. Indeed, Courtine et al. (2008) demonstrated that propriospinal neurons can bypass staggered hemisections at T7 and T12 to facilitate supraspinal stepping control in mice. Corticospinal tract axons can also sprout collaterals onto propriospinal neurons following an incomplete rodent SCI (Bareyre et al., 2004). While short connections were lost, corticospinal connections to long axons crossing the lesion were functional 12 weeks post-SCI. Thus, electrical stimulation may also modulate propriospinal networks to promote supraspinal communication below the injury site (Harkema et al., 2011; Eisdorfer et al., 2020).

Although current evidence is insufficient, electrical stimulation may promote axonal regeneration across the injury site. Udina et al. (2008) evaluated the effect of PNS on the outgrowth of ascending central sensory neuron projections across a T8 dorsal column transection model. One hour of PNS (sciatic nerve, 20 hz) immediately following injury resulted in significant regeneration of axons into the lesion site (Udina et al., 2008). Similarly, Goganau et al. (2018) also demonstrated that 1 h of sciatic nerve stimulation (20 Hz) could promote axonal outgrowth after a C4 dorsal column transection. However, both studies found that the axonal outgrowth failed to bridge the lesion site entirely, and the regenerative effects of electrical stimulation were less than that induced by lesioning the sciatic nerve pre-SCI (pre-conditioning effects) (Udina et al., 2008). Furthermore, axonal regeneration did not correlate with improvements in thermal hyperalgesia or mechanical allodynia (Goganau et al., 2018). Both studies were limited in that motor function was not analyzed, so the importance of PNS-induced axonal regeneration on motor output requires clarification.

For EES, axonal regeneration across the injury site requires further evaluation; however, combinatorial approaches have demonstrated regenerative effects. A complete rodent T7/T8 transection SCI study demonstrated that EES and inclined grid rehabilitation combined with a transplant of olfactory ensheathing cells resulted in significant axonal growth into the injury site (Thornton et al., 2018). Thus, electrical stimulation may promote axonal regeneration, particularly when used in combinatorial treatments. However, further research is required to fully elucidate whether axonal regeneration across the injury site occurs with electrical stimulation and whether this accounts for functional improvements. In addition, these studies all utilized transection models of SCI that, while appropriate for examining axonal regeneration, lack clinical relevance. Exploring axonal regeneration within contusion SCI models is necessary to strengthen the evidence for axonal regeneration after electrical stimulation.

Considering these findings together, it is likely that electrical stimulation immediately engages SC^*Vsx*2:*Hoxa*10^ neurons via large-diameter afferent projections, which may promote immediate functional recovery. SC^*Vsx*2:*Hoxa*10^ neurons may then undergo local neuroplasticity, increasing synaptic density onto motoneurons. When stimulation is paired with rehabilitation, reticulospinal and propriospinal descending pathways likely undergo neuroplastic remodeling onto these SC^*Vsx*2:*Hoxa*10^ neurons. Axonal regeneration may also contribute, but the evidence is currently lacking. This new, functionally active, descending pathway may facilitate supraspinal control that persists without electrical stimulation, ultimately improving function.

### 4.3. Upregulation of neurotrophic factors

Spinal cord remodeling via electrical stimulation may be due to an upregulation of neurotrophic factors. Jin et al. (2002) showed that ReST axons extend significantly further into a lesion site transplanted with modified brain-derived neurotrophic factor (BDNF) secreting fibroblasts compared to those transplanted with unmodified fibroblasts. Hence, increased BDNF expression post-SCI may contribute to ReST neuroplasticity and improved outcomes. Several studies have described upregulated neurotrophic factors following electrical stimulation. In a rodent T10 contusion SCI model, significant elevations in BDNF were observed after EES (100 hz, 1 h daily, 14 days) (Ghorbani et al., 2020). This study is limited in that results were obtained solely from immunoblotting, and that behavioral outcomes were not presented. However, findings by Hayashi et al. (2019) support the importance of BDNF for improving functional outcomes. Percutaneous stimulation (5 days weekly for 4 weeks) significantly increased BDNF-positive cells within the lesion site, and was associated with improved BBB open field and inclined plane scores and decreased cavity volume (Hayashi et al., 2019). PNS may have similar effects, with stimulation (sciatic nerve, 1 h, 20 hz) following a sciatic nerve lesion significantly increasing BDNF expression within anterior horn spinal cord neurons (Wenjin et al., 2011). Thus, electrical stimulation may upregulate BDNF expression, which may promote neuroplasticity and improved outcomes.

Increased BDNF expression may promote spinal cord remodeling through several signaling pathways. After release, BDNF primarily binds to the TrkB receptor to exert downstream cellular effects. Brief PNS (1 h, 20 Hz) can upregulate both BDNF and TrkB mRNA expression following femoral nerve transection, suggesting that electrical stimulation promotes this signaling pathway (Al-Majed et al., 2000). The BDNF/TrkB cascade has three main signaling pathways beneficial to neuronal function (Keefe et al., 2017). The activation of the phosphatidylinositol-3 kinase (PI-3 kinase) and Akt pathway is associated with pro-survival signals within neurons. Alternatively, BDNF can promote the phospholipase C (PLC) signaling pathway that results in calcium signaling and influences synaptic plasticity (Weishaupt et al., 2012). Lastly, BDNF can promote the upregulation of extracellular signal-regulated kinase (ERK), which has an inhibitory effect on phosphodiesterase-4 (PDE4) that typically breaks down cAMP. Thus, the upregulation of BDNF can indirectly increase cAMP levels (Weishaupt et al., 2012).

The cAMP is a secondary messenger that may contribute to axonal regeneration and neuroplasticity (Guijarro-Belmar et al., 2019). Some evidence suggests that electrical stimulation can increase cAMP expression post-SCI. Indeed, 1 h of PNS of the sciatic nerve (20 Hz) can also promote axonal outgrowth in dorsal column transection models (Udina et al., 2008; Goganau et al., 2018). Udina et al. (2008) noted that axonal outgrowth was associated with a significant increase in cAMP in the lumbar dorsal root ganglion. Thus, electrical stimulation may promote axonal outgrowth and spinal cord remodeling through mechanisms involving both the BDNF and cAMP signaling pathways.

**FIGURE 6:**
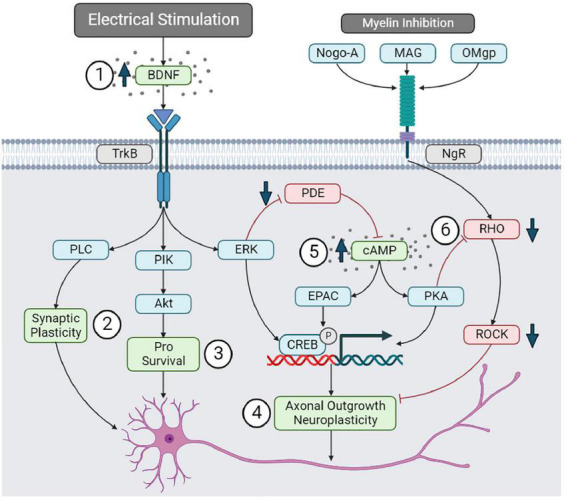
Brain-derived neurotrophic factor and cAMP signaling pathways. (1) Electrical stimulation may upregulate BDNF, promoting (2) synaptic plasticity through the PLC pathway, (3) neuronal survival through the PIK-Akt pathway, and (4) axonal outgrowth through EKR and pCREB. (5) BDNF can upregulate cAMP by inhibiting PDE. cAMP may promote neuroplasticity by upregulating pCREB. (6) cAMP can also reduce myelin inhibition of axonal outgrowth by inhibiting the RHO-ROCK pathway.

Similarly to BDNF, cAMP may influence spinal cord remodeling through multiple downstream signaling cascades. cAMP activates PKA, which can initiate the transcription of proteins that promotes axonal outgrowth. Increased cAMP expression may overcome the inhibitory role of myelin-associated proteins (Nogo-A, MAG, and OMgp) that typically restrict axonal outgrowth (Hannila and Filbin, 2008). Myelin-associated inhibitors primarily act on the Nogo-receptor (NgR) and limit axonal outgrowth through the RHO-ROCK signal cascade. The activation of PKA by cAMP has an inhibitory effect on RHO, limiting myelin’s inhibition of axonal outgrowth (Hannila and Filbin, 2008). Therefore, the BDNF and cAMP downstream processes may reduce myelin inhibition and promote the transcription of growth factors that facilitate spinal cord remodeling following electrical stimulation (Figure 6).

While this pathway may promote neuroplasticity and improve outcomes, some evidence suggests that increased p-CREB via the BDNF and cAMP pathways can contribute to neuropathic pain. Indeed, pERK and pCREB expression correlated with developing at-level mechanical allodynia following a rodent T10 contusion SCI (MASCIS injury device, 12.5 mm weight drop) (Crown et al., 2006). This raises an interesting question: if electrical stimulation upregulates pCREB, which is associated with neuropathic pain development, then how can electrical stimulation also cause neuropathic pain relief? Interestingly, Matsuo et al. (2014) examined transcutaneous stimulation (100 hz) over the left L1–L6 spinal cord levels in a rodent neuropathic pain model (left hind limb spared nerve injury). Stimulation immediately following the injury improved mechanical and thermal hyperalgesia, and significantly decreased p-CREB expression in dorsal horn neurons (Matsuo et al., 2014). This finding contradicts the above neuroplasticity mechanisms via BDNF and cAMP upregulation following electrical stimulation in SCI studies. A possible explanation for this is that electrical stimulation may cause neuropathic pain relief through alternative mechanisms that do not impede neuroplastic remodeling. In the same study, Matsuo et al. (2014) observed a significant decrease in microglia and astrocyte activation in the lumbar dorsal horn, and significantly reduced pro-inflammatory cytokine expression. Furthermore, the administration of naloxone (an opioid receptor antagonist) before stimulation prevented the downregulation of microglia and astrocytes, ultimately inhibiting neuropathic pain relief (Matsuo et al., 2014). Thus, decreased pCREB in dorsal horn neurons may reflect reduced neuronal hyperexcitability via modulated neuroinflammation within this region. Further research into pCREB, neuropathic pain, and electrical stimulation in SCI models is warranted; however, neuroinflammatory processes may play a significant role.

### 4.4. Modulation of glial cells and neuroinflammation

Glial cells exert several roles within the spinal cord and are critical for supporting neuronal functions. Following SCI, glial cells significantly influence the secondary injury cascade and are a clear target for intervention (Alizadeh et al., 2019). Notably, oligodendrocyte cell death post-SCI contributes to demyelination and improper axon conduction. Microglia, astrocytes, and peripheral immune cells also contribute to the vast neuroinflammatory response that can expand tissue damage and worsen outcomes (Oyinbo, 2011). Further, microglia and astrocytes are two components in the tetrapartite synapse model, suggesting their role in neuropathic pain (De Leo et al., 2006). Electrical stimulation may modulate myelination and neuroinflammation to improve outcomes post-SCI.

#### 4.4.1. Oligodendrocytes and myelination

Appropriate myelination is necessary for signal transduction and functional recovery. Some evidence suggests that electrical stimulation may enhance oligodendrocyte survival and reduce myelin loss post-SCI. Li et al. (2020) recently examined the effect of EES (50 Hz) on oligodendrocytes following a rat T10 contusion SCI. Compared to SCI-only animals, electrical stimulation significantly increased luxol fast blue positive area and myelin basic protein expression, reduced TUNEL staining for apoptotic cells within the white matter, and significantly increased mRNA protein levels of CNPase – an enzyme expressed by oligodendrocytes. This was associated with a significant improvement in BBB open-field score (Li et al., 2020).

The FES therapy may also promote the proliferation of oligodendrocytes within the lumbar spinal cord. Becker et al. (2010) provided FES therapy (1 h, three times daily) over the peroneal nerves 3 weeks after a T9 suction ablation injury. They noted a significant increase in Brd-U labeled progenitor cells in the lumbar spinal cord at 36 days post-SCI. At 43 days post-SCI, there was a decrease in progenitor cells and an increase in newborn APC+ oligodendrocytes. The authors suggested that FES therapy may have promoted the maturation of the progenitor cells into oligodendrocytes within the lumbar spinal cord (Becker et al., 2010). PNS also appears to influence oligodendrocytes. In a rodent multiple sclerosis model (T12 unilateral focal demyelination of the dorsal columns), PNS (sciatic nerve, 20 hz) significantly increased myelin basic protein expression and oligodendrocyte numbers (Ayanwuyi et al., 2022). While this study demonstrates that PNS can influence myelination, this finding must be reproduced within SCI models to confirm it is a potential mechanism for PNS. Nonetheless, there is evidence that electrical stimulation may enhance oligodendrocyte numbers and myelination (Li et al., 2020).

Promoting oligodendrocyte survival/differentiation and myelination is generally considered beneficial post-SCI. Demyelinated axons have impaired signal transduction, impacting function and making axons susceptible to further damage by ionic imbalances (Plemel et al., 2014). Thus, enhancing oligodendrocyte numbers and myelination may contribute to functional improvements.

#### 4.4.2. Modulation of neuroinflammation

Electrical stimulation may also influence the neuroinflammatory response. Ayanwuyi et al. (2022) observed that sciatic nerve stimulation could modulate macrophage/microglia polarization in a T12 dorsal column demyelination model. ED-1 positive macrophage/microglia within the spinal cord exhibited significantly greater expression of the pro-repair molecule Arginase-1 and a significant decrease in TNF-α, a cytokine typically considered pro-inflammatory (Ayanwuyi et al., 2022). Thus, electrical stimulation may also modulate the neuroinflammatory response to promote tissue survival post-SCI. Further supporting this, a single 30-min session of bilateral, high-frequency transcutaneous electrical nerve stimulation (100 hz at 90% of motor threshold) 5 weeks following a severe T12 contusion SCI (50 mm contusion, NYU impactor) significantly reduced microglial activation in the dorsal and ventral gray matter regions. This was associated with a temporary decrease in spasticity up to 40 min following treatment (Hahm et al., 2014).

Electrical stimulation may also influence circulating concentrations of inflammatory cytokines. After 10 weeks of FES cycling activity in 18 patients with SCI, plasma levels of CRP, IL-6, and TNF-α significantly decreased. This was associated with an increased muscle mass and significant improvement in motor and sensory function (Griffin et al., 2009). Additionally, Bakkum et al. (2015) found significant decreases in CRP and IL-6 concentrations and IL-6/IL-10 ratio in patients with chronic SCI (>10 years post-injury) after either 16-weeks FES hybrid cycling exercise regime or hand-cycling exercise without FES. However, there was no significant difference between FES cycling and hand-cycling alone cohorts (Bakkum et al., 2015). While these studies are limited in that they cannot identify inflammatory changes within the spinal cord itself, they do suggest that electrical stimulation may impact inflammation in a clinical population.

While the effect of electrical stimulation on neuroinflammatory mechanisms has not received significant attention post-SCI, neuropathic pain studies may provide insight. In 12 patients with failed back surgery syndrome who received burst spinal cord stimulation (40 Hz burst rate and 500 Hz intra-burst rate), a significant decrease in visual analog scale score for back pain correlated with a significant increase in serum IL-10 concentrations (Kinfe et al., 2017). Two hours following an L5 spinal nerve root ligation, sciatic nerve stimulation at 2 Hz or 20 Hz alleviated mechanical and thermal hypersensitivity. This was associated with a significant decrease in Iba-1 (microglia) and GFAP (astrocytes) density and decreased spinal cord inflammatory cytokines (IL-1β, IL-6, and TNF-α) at 7 days post-injury (Wong et al., 2022).

Stephens et al. (2018) examined spinal cord stimulation (50 Hz, 80% motor threshold, 0.2 ms, and 120 min/session) following a left sciatic nerve chronic constrictive injury in rodents. Using RNA-seq analysis in the spinal cord tissue, they noted that electrical stimulation upregulated genes involved with inflammatory processes (Stephens et al., 2018). Similarly, Sivanesan et al. (2019) utilized RNA-seq analysis of spinal cords after stimulation in a paclitaxel-induced peripheral neuropathy model. Analysis revealed that SCS upregulated genes associated with the inflammatory and immune response, with both astrocyte and microglia associated genes enhanced compared to sham stimulation (Sivanesan et al., 2019). Unfortunately, the study did not further elucidate the physiological function of these genes. However, more recent transcriptome analysis after spinal cord stimulation has revealed these functions in greater detail. Vallejo et al. (2020) also used RNA-seq on spinal cord tissue following spared nerve injury, and found that high rate (1,200 Hz and 50 μs PW) and differential targeted multiplex programming (DTMP, pulsed signals between 20 and 1,200 Hz and max PW of 500 μs) significantly decreased GFAP (astrocytes) expression. DTMP stimulation also significantly modulated *C1qa* (involved in synaptic pruning), *Casp1* (encodes an enzyme that catalyzes the formation of pro-inflammatory cytokines) and *Tal1* (regulates phenotyping of microglia toward a neurotoxic state) toward naïve levels (Vallejo et al., 2020). These results utilizing advanced analysis techniques seem to support neuroinflammation findings from SCI models, but further research into transcriptome changes following SCI and electrical stimulation is warranted to confirm these similarities.

Such results suggest that electrical stimulation modulates neuroinflammation toward a response typically considered anti-inflammatory/pro-repair. While some studies have observed improved motor function by modulating neuroinflammation, this mechanism seems particularly relevant for neuropathic pain relief. Further research within SCI models is required to establish further the effects of electrical stimulation on neuropathic pain post-SCI.

### 4.5. Summary of cellular and molecular mechanisms

**FIGURE 7:**
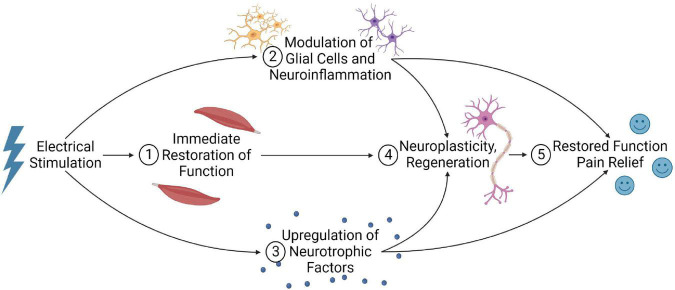
Summary of the potential mechanisms that electrical stimulation may improve outcomes post-SCI. (1) Electrical stimulation can immediately restore function by activating local neural circuitry and facilitating spared, residual supraspinal inputs to regain functional control below the lesion. (2) Stimulation may also modulate glial cells and neuroinflammation, and (3) upregulate neurotrophic factors. (4) When combined with long-term stimulation and rehabilitation, this may promote neuroplastic remodeling of the spinal cord and possibly axonal regeneration, facilitating supraspinal control below the injury. (5) These mechanisms may account for the restored function and neuropathic pain relief observed in electrical stimulation studies.

Due to the complicated pathophysiology of SCI, several mechanisms contribute to improved outcomes with electrical stimulation. These mechanisms likely have synergistic effects to enhance the spinal cord micro-environment and promote functional recovery (Figure 7).

## 5. Potential alternative mechanisms

While the above mechanisms play a significant role, several alternative mechanisms may also contribute to improved outcomes but have received less attention. Recently, Griffin and Bradke (2020) identified seven mechanisms for therapeutic interventions post-SCI, including: (1) reducing secondary damage, (2) replacing lost cells, (3) removing inhibitory molecules, (4) regeneration, (5) resupplying trophic support, (6) remyelination, and (7) rehabilitation. These targets can be compared to our mechanistic understanding of electrical stimulation to identify gaps in current knowledge. Evidence in Section “4. Cellular and molecular mechanisms of electrical stimulation” suggests electrical stimulation may improve function through mechanisms 4–7. However, evidence that electrical stimulation reduces secondary damage, replaces lost cells, and removes inhibitory molecules is lacking. These targets present alternative mechanisms that may also contribute to improved outcomes.

### 5.1. Reduction of secondary damage

Reducing the extent of secondary damage may decrease lesion size and preserve function. Electrical stimulation may reduce secondary damage by modulating glial cells and neuroinflammation (see section “4.4. Modulation of glial cells and neuroinflammation”). Indeed, EES and PNS can reduce white matter apoptotic cells 7 days post-SCI (Hayashi et al., 2019; Li et al., 2020). However, myelination and neuroinflammation present only a small portion of the multifaceted secondary injury cascade. Ischemia, hemorrhage, and BSCB permeability play an extensive role in secondary tissue damage post-SCI and are therapeutic targets (Ahuja et al., 2017). Yet, these acute and subacute processes have received little attention. Examining these events may help elucidate if electrical stimulation can further reduce secondary damage via an alternative mechanism to neuroinflammation. While understudied for SCI, some stroke research suggests that electrical stimulation may have vascular consequences. Indeed, transcranial direct current stimulation after ischemic stroke can enhance blood-brain-barrier permeability, thus potentially worsening outcomes (Peruzzotti-Jametti et al., 2013). Possibly, acute electrical stimulation post-SCI may reduce BSCB integrity in a similar manner, which may impact outcomes and present a barrier to early stimulation. Nonetheless, this requires evaluation within SCI models to confirm or refute.

The time point of stimulation in current studies may have limited our understanding of this mechanism Electrical stimulation studies often incorporate long-term rehabilitation protocols in individuals with chronic SCI (Table 1); hence, many preclinical studies mimic this and analyze mechanisms at later time points. However, differences in the spinal cord microenvironment at acute and chronic stages of SCI likely influence cellular and molecular mechanisms. Delivering stimulation acutely post-SCI may influence secondary injury processes at their peak to reduce secondary tissue damage and preserve function. Possible barriers to providing electrical stimulation acutely post-SCI must be considered, particularly for invasive devices that require surgical implantation. Nonetheless, the potential benefits of reducing tissue damage make exploring acute electrical stimulation a worthwhile investigation.

### 5.2. Replacement of lost cells

The SCI causes significant cell death; hence, replacing lost cells within the lesion site may create a growth-permissive environment that facilitates regeneration and improves function (Griffin and Bradke, 2020). There is limited evidence that electrical stimulation replaces lost cells within the injury site. While EES, FES, and PNS can increase oligodendrocyte numbers within the spinal cord (see section “4.4.1. Oligodendrocytes and myelination”), this was not necessarily localized within the lesion site. For example, Becker et al. (2010) observed remyelination in the lumbar cord after FES in a T9 SCI but did not investigate remyelination at the injury site. Hence, electrical stimulation may replace lost cells, but perhaps not within the lesion. Since neuroplasticity is a key mechanism, electrical stimulation may predominately influence tissue around the lesion rather than replacing cells within it. Nonetheless, future research examining cell birth and repopulation within the injury site may be needed to elucidate this mechanism.

### 5.3. Removal of inhibitory molecules

Inhibitory molecules are present within the glial scar in the chronically injured spinal cord, presenting a barrier to axonal regeneration into and across the lesion site. Electrical stimulation can overcome myelin-associated inhibition through the cAMP downstream pathway (see section “4.3. Upregulation of neurotrophic factors”). However, targeting extracellular matrix proteins (CSPG, tenascin, and NG2 proteoglycan) has received little attention. Al’joboori et al. (2020) examined the effect of EES and locomotor training on CPSG expression following a T9/T10 complete transection SCI, but did not find an effect of stimulation. However, this study was limited by a short training time under stimulation (30 min per day, 5 days per week) and the use of a complete spinal cord transection rather than contusion injury model. Furthermore, the study examined CSPG expression in the lumbar spinal cord rather than at the injury site. Interestingly, the sustained delivery of BDNF to the lesion cavity following SCI can reduce CSPG deposition and promote axonal outgrowth (Jain et al., 2011). As electrical stimulation can also upregulate BDNF, extracellular matrix proteins may be similarly influenced. This may facilitate the axonal outgrowth into the lesion observed in some studies (see section “4.2.2. Reorganization of descending pathways”), although evidence for this is limited. Further research into the effects of electrical stimulation on the extracellular matrix is warranted.

### 5.4. Summary of alternative mechanisms

**FIGURE 8:**
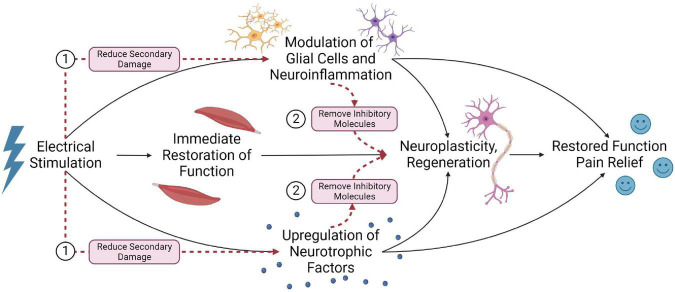
Alternative mechanisms that may contribute to functional recovery post SCI. (1) Providing acute electrical stimulation may reduce secondary damage through neuroinflammatory and vascular events. (2) Electrical stimulation may remove inhibitory molecules around the lesion site to facilitate regeneration.

Several alternative mechanisms may contribute to functional recovery but require further evaluation. Electrical stimulation may have acute effects that reduce secondary damage and may overcome inhibitory molecules at the lesion site. This may occur concurrently with pre-established mechanisms of electrical stimulation to restore function (Figure 8). Evidence that electrical stimulation replaces lost cells at the lesion site is limited and requires further investigation.

### 5.5. Combinatorial strategies

The complicated pathophysiology of SCI suggests that a combinatorial strategy may be necessary. This may be achieved by combining treatments that work by similar mechanisms to those engaged by electrical stimulation. For example, serotonin receptor agonists can facilitate lumbosacral CPG networks to promote locomotion, similar to electrical stimulation (Ghosh and Pearse, 2015). Asboth et al. (2018) combined EES with serotonergic and dopaminergic receptor agonists (electrochemical neuromodulation) following a severe T10 rodent contusion SCI. 100% of rats that received electrochemical neuromodulation regained weight-bearing locomotion compared to 88% of EES-only rats (Asboth et al., 2018). Hence, exploring combinatorial treatments that engage similar mechanisms to electrical stimulation may have synergistic effects and improve outcomes.

Alternatively, combinatorial treatments may target mechanisms not engaged by electrical stimulation. For example, replacing lost cells is a mechanism potentially not influenced by electrical stimulation (see section “5.2. Replacement of lost cells”). Hence, combinatorial treatments may be necessary. Fadeev et al. (2021) piloted a combination of EES and triple gene therapy following a T8 rodent contusion SCI. The triple gene therapy involved an intrathecal injection of gene-engendered human umbilical cord blood mononuclear cells expressing VEGF, GDNF, and NCAM. This significantly increased gray matter sparing and synaptophysin (a synaptic marker) immunoreactivity compared to EES alone (Fadeev et al., 2021). Although limited by sample size and control groups, Islamov et al. (2020) showed similar positive results of EES and triple gene therapy in a porcine SCI model. In another approach, EES combined with olfactory ensheathing cells or fibroblast transplants at the injury site can promote axonal regeneration, but functional outcomes are unclear (Thornton et al., 2018). Extensive research is required to establish the efficacy of these combinatorial treatments, but these positive results highlight the potential of these strategies to improve outcomes.

## 6. Considerations for future studies

Future studies must consider several factors in their design, including analysis methods, research models, stimulation parameters and potential barriers. These factors are essential to improve our mechanistic understanding and translate electrical stimulation interventions into clinical settings.

### 6.1. Study methods, models, and stimulation parameters

Utilizing appropriate analysis methods is vital for determining cellular and molecular mechanisms. Many preclinical studies use immunoblotting, immunohistology, and tract-tracing techniques (Table 1). However, recent advancements in transcriptomic analysis techniques provide an exciting opportunity to explore the mechanisms of electrical stimulation in greater detail. While RNA-seq analysis has been used following electrical stimulation in neuropathic pain models (Stephens et al., 2018; Sivanesan et al., 2019; Vallejo et al., 2020), transcriptome analysis has seen limited uses with electrical stimulation following SCI. A notable application was by Kathe et al. (2022), who recently used spatial transcriptomics and single-nucleus RNA-seq after EES and SCI. This provided significant mechanistic insight by highlighting neurons critical for recovering locomotion. Outside of this study, however, there have been very few SCI electrical stimulation studies that have incorporated transcriptome analysis. Further utilization of these techniques may help consolidate our current understanding or elucidate novel mechanisms.

Similarly, clinical studies primarily use electrophysiology, neurological exams and immunoassays of blood samples (Table 1). Incorporating alternative techniques into clinical trials may provide further mechanistic insight. Notably, Kathe et al. (2022) used positron emission tomography (PET) with ^18^F-fluorodeoxyglucose (^18^F-FDG-PET), which revealed decreased metabolic activity from EES within human participants. Other studies using PET to analyze mechanisms of electrical stimulation are limited, but future studies could incorporate this technique. For example, PET and the 18-kDa translocator protein (TSPO) could be used to monitor neuroinflammation in clinical electrical stimulation studies (Tremoleda et al., 2016). MRI techniques may also provide mechanistic insight, although device compatibility must be considered. Previously, fMRI has been used to study neuronal activation during transcutaneous stimulation in individuals with SCI (Zhong et al., 2017). Further incorporation of fMRI into electrical stimulation studies may help identify neurological regions that are targeted by stimulation and monitor changes in activation over time. Diffusion tensor imaging (DTI) has also been recommended for use following FES to measure spinal cord integrity (Mulcahey et al., 2012). While applications with electrical stimulation after SCI are limited, stroke studies of FES have incorporated DTI to analyze corticospinal tract integrity (Zheng et al., 2018). Similar applications following SCI may further elucidate the neuroplastic or regenerative effects of electrical stimulation. Imaging techniques represent opportunities to further gain mechanistic insight, quantify functional improvements, and potentially inform and guide electrical stimulation applications within clinical settings. Hence, greater utilization of these techniques in future clinical studies would be beneficial.

Future preclinical studies of electrical stimulation mechanisms should carefully consider the appropriate SCI model. In a recent systematic review, Sharif-Alhoseini et al. (2017) determined that most SCI studies utilize rat (72.4%) or mouse (16%) models. Despite having several advantages, including accessibility, low cost, and the ability to genetically modify animals, these models may not be ideal for electrical stimulation research. This was highlighted by Formento et al. (2018) when they discovered that antidromic action potentials elicited by EES can collide with natural proprioceptive signals. Interestingly, these collisions occur within humans but not in rats (Formento et al., 2018), emphasizing the need for careful SCI model selection.

Lumbosacral spinal cord structure also differs between species, potentially impacting electrical stimulation studies that target this region. In a cross-species comparison, the lumbosacral spinal cord of humans is most similar in size and morphology to the domestic pig, closely followed by monkeys and cats (Toossi et al., 2021). Functional anatomy is also more similar in the domestic pig, with the corticospinal tract showing greater similarity to humans compared to that of rodents (Leonard et al., 2017). This has mechanistic implications, as the neuroplastic remodeling of descending pathways contributes to recovery with electrical stimulation. Domestic pig SCI models have been developed (Gayen et al., 2022), along with various alternative porcine models that are well-characterized (Kim et al., 2018; Weber-Levine et al., 2022). Alternatively, non-human primate models of SCI offer an attractive option for mechanistic research due to their similar genetic, biological and physiological properties to humans (Nardone et al., 2017). While the costs, extensive animal care requirements, and ethical considerations present an accessibility barrier (Sharif-Alhoseini et al., 2017), several studies have successfully utilized porcine or primate models for electrical stimulation research (Capogrosso et al., 2016; Fadeev et al., 2020; Greiner et al., 2021). Greater utilization of these models may be necessary to understand how mechanisms established in small animal models compare to human SCI, to facilitate clinical translation.

Along with species, the modality of SCI requires consideration. Many preclinical electrical stimulation studies utilize spinal cord transection models that, while useful for evaluating axonal regeneration post-SCI, do not accurately model the contusion-compression injury characteristic observed in most human injuries (Asboth et al., 2018). While transection models completely cut off supraspinal centers, contusion injuries preserve a ring of tissue that contains residual ascending and descending fibers – similar to observations in human SCI (Sherwood et al., 1992). This likely has mechanistic implications, since remodeling of residual pathways that extend within this spared tissue is a key mechanism, while evidence for axonal regeneration is limited. Additionally, contusion models closely replicate pathophysiological processes post-SCI, making them a preferred model for evaluating secondary injury events (Sharif-Alhoseini et al., 2017). While both transection and contusion SCI models may be needed to research different processes, this should be carefully considered and justifiable by the hypothesized mechanisms.

Stimulation parameters must also be thoroughly researched, as different stimulation protocols can alter mechanisms and outcomes. Indeed, Tang et al. (2020) observed a positive correlation between hemodynamic and electromyography responses at low frequencies (20 – 40 Hz) compared to high frequencies (200 – 500 Hz). Additionally, stimulation of the sciatic nerve at 2 and 20 Hz can reduce neuropathic pain and neuroinflammation following a rodent L5 nerve root ligation, while 60 Hz was deemed ineffective (Wong et al., 2022). Interestingly, there may be time points post-SCI that benefit from different stimulation parameters. For example, certain stimulation parameters can increase blood flow, which would be detrimental acutely post-SCI when the BSCB is permeable. However, once the BSCB is re-established, stimulation parameters could be altered to increase blood flow and improve tissue perfusion. Given current limitations in mechanistic understandings, it may be premature to incorporate this complexity into study designs. However, treatment strategies that alter stimulation paradigms over time to target specific mechanisms may improve efficacy.

### 6.2. Challenges associated with electrical stimulation

There are several challenges to consider before electrical stimulation devices are more widely adopted. Firstly, these devices must prove cost-effective to become readily available. While there is limited evidence for SCI, studies of failed back surgery syndrome suggest that EES is cost-effective. Despite higher initial costs due to the device implantation, the break-even point was approximately 24 months post-implant, and the estimated cost/quality of life year gained ratio was below the $25,000 cut-off that insurers are willing to pay (McClure et al., 2021). Hence, it is likely that electrical stimulation devices are also cost-effective for SCI, particularly for less invasive devices. Nonetheless, this will require formal evaluation for each stimulation modality before wider clinical adoption.

Potential barriers to the clinical use of electrical stimulation must also be considered. In a survey of 298 physical therapists, barriers to utilizing FES for stroke patients included a lack of resources and training for FES, client characteristics, and the therapist’s treatment preference (Auchstaetter et al., 2016). For EES, a survey of 42 physicians suggested that additional research is needed to show efficacy, that clear guidelines on stimulation parameters are lacking, and that there is a lack of knowledge on which patients will benefit (Solinsky et al., 2020). Several of these concerns are notable within mechanistic electrical stimulation studies, including a lack of diversity in clinical trial participants and variability in stimulation parameters (Table 1). Developing strategies to overcome these barriers will be essential for the wider adoption of electrical stimulation post-SCI. Expanding the scope of clinical studies to include broader demographics may better inform clinicians as to which patients will benefit.

The duration of improved outcomes with electrical stimulation may also present a barrier. Some studies have reported that improvements are temporary after ceasing stimulation. For example, Hahm et al. (2014) found that reduced spasticity via PNS was only maintained for 20–50 min in a rodent SCI model. Similarly, Stephens et al. (2018) found that neuropathic pain returned to pre-stimulation levels after 30 min of ceasing EES in a rodent pain model. While EES and FES have both reported improvements to motor function that persist beyond stimulation, the duration that these improvements can be maintained without recommencing stimulation is unclear. Characterizing the duration of improved outcomes without stimulation may not be a priority for devices that deliver long-term stimulation continuously to permit function. However, better understanding the longevity of improvements is necessary if electrical stimulation is to be used temporarily for therapeutic purposes. In particular, this would benefit future studies by allowing stimulation protocols to be optimized such that stimulation could be delivered at the appropriate time to maintain functional improvements.

Most importantly, the perspectives of individuals with SCI must be incorporated. Previously, it has been suggested that some individuals with SCI may refuse to receive implant devices, as having an implanted device may exclude them from future clinical trials of potentially more effective treatments (Adams et al., 2007). Individuals with SCI may have better perceptions of non-invasive stimulation modalities. Houston et al. (2021) reported feedback from five individuals with chronic SCI who participated in a surface-electrode FES and visual feedback training program. These individuals reported more confidence in their balance, but also stated that they were wary of being over-confident in their improvements. Nonetheless, these individuals stated that the treatment had led to improvements in daily living (Houston et al., 2021), suggesting that this type of intervention may be perceived as beneficial for individuals with SCI. These opinions provide valuable insight that must be considered as we continue to optimize electrical stimulation therapies.

### 6.3. Differences between stimulation modalities

In this review, we have examined the combined mechanisms of EES, PNS, and FES rather than evaluating the interventions individually. This was done for two reasons: firstly, as EES, PNS, and FES primarily stimulate similar afferent fibers, there is likely an extent of overlap between the cellular and molecular mechanisms (Duffell and Donaldson, 2020). Secondly, this review provides a broad summary of potential mechanisms. Given the paucity of current mechanistic understandings, combining multiple stimulation modalities allowed us to suggest several mechanisms for future consideration.

While there are similar mechanisms, differences between these stimulation modalities do exist. Notably, EES likely recruits afferents across more spinal levels compared to PNS or FES (Duffell and Donaldson, 2020). This broader activation allows greater specificity with EES, facilitating highly coordinated movements across several tasks (Rowald et al., 2022). This is advantageous to FES and PNS, which stimulate specific nerves necessary for a targeted outcome. Further, EES and FES incorporate descending drive from the participant to control musculature, which is absent in PNS studies. Thus, EES and FES may promote greater descending neuroplasticity, as these are directly involved in the therapy. As a result, there may be different mechanisms engaged between stimulation devices. Furthermore, the wide range of FES and PNS techniques warrants further investigation. Comparing different electrode designs may be necessary to establish if mechanisms are congruent between electrode designs.

Finally, we have only examined stimulation modalities that target the nervous system below the injury site. Other techniques that are outside this review’s scope, such as transcranial magnetic stimulation and deep brain stimulation, may provide mechanistic insight and are worth considering in future research.

## 7. Conclusion

The prospects of electrical stimulation following SCI are compelling, having shown promising preliminary results in clinical trials. While the cellular and molecular mechanisms remain to be fully elucidated, neuroplastic remodeling of the spinal cord, the upregulation of neurotrophic growth factors, remyelination, and the modulation of neuroinflammation appear to contribute. These mechanisms likely work in tandem with each other to improve patient functions and reduce pain. Further research is required to consolidate our understanding and examine potential alternative mechanisms that may contribute. Nonetheless, EES, FES, and PNS have significant potential, and with the speed of advancements, it is an exciting time for SCI research.

## Author contributions

RD and AVL conceived of and designed the review. RD performed the review of the literature and drafted the manuscript. CB, AL, and AVL reviewed and edited the manuscript. All authors read and approved the final manuscript.
